# Physics-Based Differentiable Rendering for Efficient and Plausible Fluid Modeling from Monocular Video

**DOI:** 10.3390/e25091348

**Published:** 2023-09-17

**Authors:** Yunchi Cen, Qifan Zhang, Xiaohui Liang

**Affiliations:** School of Computer Science and Engineering, Beihang University, Beijing 100191, China; cenyc@buaa.edu.cn (Y.C.); zhangqf_m@buaa.edu.cn (Q.Z.)

**Keywords:** monocular video, fluid reconstruction, differentiable renderer

## Abstract

Realistic fluid models play an important role in computer graphics applications. However, efficiently reconstructing volumetric fluid flows from monocular videos remains challenging. In this work, we present a novel approach for reconstructing 3D flows from monocular inputs through a physics-based differentiable renderer coupled with joint density and velocity estimation. Our primary contributions include the proposed efficient differentiable rendering framework and improved coupled density and velocity estimation strategy. Rather than relying on automatic differentiation, we derive the differential form of the radiance transfer equation under single scattering. This allows the direct computation of the radiance gradient with respect to density, yielding higher efficiency compared to prior works. To improve temporal coherence in the reconstructed flows, subsequent fluid densities are estimated via a coupled strategy that enables smooth and realistic fluid motions suitable for applications that require high efficiency. Experiments on synthetic and real-world data demonstrated our method’s capacity to reconstruct plausible volumetric flows with smooth dynamics efficiently. Comparisons to prior work on fluid motion reconstruction from monocular video revealed over 50–170x speedups across multiple resolutions.

## 1. Introduction

Realistic fluid flow reconstruction is of paramount importance for a wide range of applications, including those related to special effects in film and video, artistic design, and digital media generation. However, this task poses significant challenges. On the one hand, fluid phenomena are ubiquitous in daily life, and people have high expectations for the quality of their rendering in artistic and media contexts. However, existing methods that attempt to reconstruct fluid motion through a combination of physics-based constraints and density estimation require tens of minutes per frame for reconstruction, rendering them unsuitable for interactive rendering applications.

To reconstruct fluid motion, existing computed tomography (CT) methods (e.g., [1,2]) rely on sparse-view images and solve a least-squares problem that relates pixels and voxels using a visual hull. However, their reconstruction quality suffers as the number of views decreases, resulting in an under-determined inverse problem. Additionally, the sophisticated setups required by these methods make calibration difficult and limit their adaptability. Our method focuses on monocular video reconstruction, which enables us to use a wide variety of existing videos and capture new fluid motions with ease. Rather than solely simulating fluids from images, recent works (e.g., [3,4]) have employed a physics-based prediction scheme to add transport constraints to optimization and couple density estimation to velocity estimation. Although these physics-based priors have improved the temporal coherence of flow motion reconstruction, the time-consuming fluid density reconstruction and complex physics-based prediction scheme remain bottlenecks to reconstruction efficiency.

Differentiable rendering has been a major focus in computer graphics recently. It comprises techniques that integrate rendering into end-to-end optimization by obtaining useful derivatives of the rendering process. However, integrating differentiable rendering into fluid reconstruction frameworks poses a huge challenge due to the complexity and nonlinearity of the relation between pixel intensities and fluid density. Franz et al. [5] proposed a differentiable-rendering-based fluid reconstruction framework, where automatic differentiation (AD) is used to compute the derivatives in a ray marching process. However, the current automatic differentiation framework is typically inefficient, especially when optimizing numerous gradient parameters.

To improve the efficiency of fluid reconstruction, we propose a physics-based differentiable rendering framework that analytically computes radiance derivatives with respect to density. Unlike prior automatic differentiation techniques, which have inherent computational overhead, our method directly calculates derivatives by deriving a differential form of the radiance transfer equation under single-scattering assumptions. This analytical approach avoids redundant operations and gradient tracking, significantly accelerating fluid density optimizations compared to previous differentiable rendering techniques.

Furthermore, we couple our optimized renderer with a constrained density and velocity estimation strategy. By propagating velocities and densities across time steps while enforcing physical constraints, we achieve temporally coherent fluid reconstructions far more efficiently than the costly tomographic methods used previously. Our major contributions are summarized as follows:

- An efficient physics-based differentiable rendering framework is proposed for fluid density reconstruction, which is more efficient than previous models.
- The differential form of the Radiative Transfer Equation (RTE) based on single scattering is derived.
- The transport constraints and the differentiable rendering step are coupled for fluid density updating, achieving smooth fluid motion reconstruction.

## 2. Related Work

**Density reconstruction:** Reconstructing fluid density from 2D observations has been a popular research topic in computer graphics. Computed tomography (CT) methods, originally developed for medical applications [6], are widely used for fluid density reconstruction. Two major categories of tomographic reconstruction algorithms are available: direct and iterative methods. Direct methods rely on the analytical inverse conversion from input images to a volume, which is derived using the Fourier transform and calculus. They require dense multi-view inputs that are difficult to obtain for fluids. Therefore, iterative methods, which formulate the relationship between input images and a volume as an energy function that is minimized using iterative algorithms, are more commonly used for fluid density reconstruction.

Ihrke and Magnor [1] proposed a tomography method to reconstruct a volumetric model from multiple images of fire by minimizing the least-squares energy using the conjugate gradient method. To enhance the efficiency of reconstruction, Ihrke and Magnor [7] used an adaptive grid to reconstruct a 3D density function from its projections, which was suitable for thin smoke and flames. Atcheson et al. [8] captured 3D gas flows using the time-resolved Schlieren tomography system. Some attempts to improve sparse tomographic reconstruction have been proposed. Okabe et al. [2] used one to two input videos and augmented their tomographic approach with appearance transfer. They reconstructed an initial density volume with regular tomography and iteratively improved the density until the reconstruction satisfied additional view constraints. Eckert et al. [4] pushed the limit of the sparse tomography problem by introducing a single-view reconstruction approach for plumes. They compensated for the lack of information by using physics-based and geometric priors.

Most of these methods often required a difficult calibration step, and the reconstruction quality dropped with the decrease in the number of views. Our method focuses on reconstructing fluid density from monocular video, offering distinct advantages, including reduced costs, a drastically simplified setup, and eliminating tedious procedures like camera calibration and synchronization.

**Velocity reconstruction:** Reconstructing fluid motion from captured videos is more challenging than reconstructing volumetric density, as motion is indirectly observed by tracing temporal changes in density. Two families of techniques exist for estimating the velocity field of a fluid: tracer-based and tracer-free approaches. Tracer-based approaches introduce tracers, such as particles or dye, into the fluid, and then the fluid velocity can be retrieved by tracing these tracers. Various PIV methods, including tomographic PIV [9], synthetic aperture PIV [10], structured-light PIV [11,12,13], and plenoptic PIV [14,15], are widely used in different fields to characterize fluid flows. However, some methods require specialized hardware and complex camera setups, as seen in [9,11]. Though PIV methods are widely used, methods like [9,11] require carefully chosen particles, specialized hardware, and complex camera setups. Tracer-free approaches, like background-oriented Schlieren tomography (BOS) [16,17], use the phase change due to refractive index differences in the fluid to track fluid flow. These approaches retrieve only either the density field or the velocity field of the fluid.

Visible-light-capturing is an alternative approach for fluid motion reconstruction. Corpetti et al. [18] used optical flow with an additional divergence-free and curl smoothness prior to regularizing 2D cloud motions. Similarly, some previous works have taken divergence-free constraints into account [19,20,21], yet most of them suffer from the complexity of solving higher-order regularization terms. Recent works [4,5] have coupled fluid density updating and velocity calculation to improve the temporal coherence of flow reconstruction. Our work adopts a similar coupled computation strategy to guarantee the time evolution of the fluid flow.

**Differentiable rendering:** Differentiable rendering is a technique that allows for the computation and propagation of derivatives of scene parameters through images. This technique has a wide range of applications, including solving analysis-by-synthesis problems and training machine learning pipelines incorporating forward-rendering processes. Loper and Black [22] introduced *OpenDR*, which approximates the backward pass of the traditional mesh-based graphics pipeline and has inspired several follow-up works [23,24,25,26,27]. Kato et al. [26] proposed *Neural Renderer*, an approximate gradient for rendering a mesh that enables the integration of rendering into neural networks. Instead of approximating the backward pass, some methods approximate the rasterization of the rendering process, enabling the computation of useful gradients. Liu et al. [27] introduced *Soft Rasterizer*, which provides an accurate soft approximation of the standard rasterizer. Although these approximation methods reduce rendering time, generating photorealistic images containing complex interactions of light, geometry, and materials becomes difficult. Li et al. [28] proposed the PBDR framework, which supports global illumination. However, all the methods mentioned so far are limited to surface-based light transport differentiation.

Recently, there has been a focus on improving the efficiency of volumetric differentiable rendering. Nimier-David et al. [29] introduced the radiative backpropagation approach to differentiable rendering. They observed that differentiable rendering is equivalent to the solution of a reversed light transport problem. Although this approach does not memorize intermediate states, it also has an expensive computation time, which becomes quadratic in the number of scattering events along a light path. To address this, Vicini et al. [30] proposed the path replay backpropagation approach, which achieves a computation time that is linear in the number of scattering events and further improves the performance. Although these approaches significantly accelerate performance compared to naive automatic differentiation implementations, prior works have shown that AD methods can be efficient if carefully designed. Weiss et al. [31] proposed an efficient strategy for differentiable direct volume rendering using automatic differentiation. They demonstrated that by carefully designing the backward pass and leveraging intermediate outputs from the forward rendering, AD can achieve excellent performance for differentiating rendered images with respect to volume densities. While representing an important advancement in efficient automatic differentiation for volumetric data, calculating derivatives via AD still fundamentally relies on propagating gradients through long computational graphs. This carries inherent overhead compared to directly computing derivatives analytically. Our work builds upon these insights by deriving an analytical form of the radiance transfer equation, enabling the direct and efficient computation of radiance derivatives with respect to density. By avoiding gradient tracking and redundant operations, our proposed approach aims to further improve performance for optimizing dynamic volumetric densities, such as in fluid reconstruction tasks.

## 3. Methods

### 3.1. Overview

**Figure 1:**
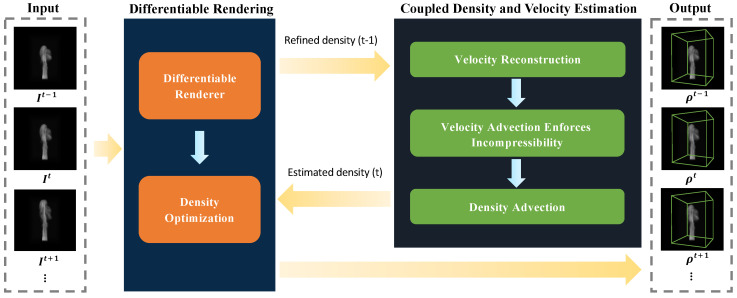
Our framework consists of two primary algorithm components: the **differentiable rendering component** and the **coupled density and velocity estimation component**. In this diagram, yellow and blue arrows delineate the directional flow of data between external and internal modules, respectively. The **differentiable rendering component** is utilized to refine volumetric representations extracted from a temporal sequence of fluidic images. We note that the current density fields are associated with the temporal epoch *t* − 1. These outputs subsequently serve as inputs for the **coupled density and velocity estimation component**. This component initially estimates the velocity field based on the density at temporal epoch *t* − 1. It then advects this velocity field to the subsequent temporal epoch *t* while enforcing incompressibility constraints. Finally, the density field is advected in accordance with the updated velocity field. The advected density volumes, along with their corresponding input images, are inputted into the differentiable renderer for final corrections.

As illustrated in Figure 1, our computational framework is composed of two main algorithmic components: the **differentiable rendering component** (Section 3.2) and the **coupled density and velocity estimation component** (Section 3.3). The primary aim of this framework is to efficiently reconstruct a temporally coherent sequence of fluid density fields, denoted as {ρ}. This reconstruction is designed to align the density fields with the input monocular fluid images {I} while conforming to essential physical priors.

Drawing from Algorithm 1, we will now expound on how our framework operates. Initially, we take a sequence of monocular fluid images {I} as input and reconstruct the first two frames of fluid density $\rho^{1}$ and $\rho^{2}$ through the **differentiable rendering component** (Lines 2–3). Here, $I^{t}$ refers to the image at a time *t* within the input video sequences, where $\left\{t|t\in N_{0},t\leq F\right\}$ and F denotes the total number of frames in the sequence. With the obtained initial fluid density, the subsequent frames of estimated fluid density ${\tilde{\rho }}^{t}$ are then computed using the **coupled density and velocity estimation component** (Line 5), which ensures that the fluid flows adhere to the underlying physical priors. Next, given the estimated density ${\tilde{\rho }}^{t}$ as input, the final fluid density $\rho^{t}$ is corrected via our differentiable renderer using the target image $I^{t}$ (Line 6).
**Algorithm 1:** Fluid motion reconstruction
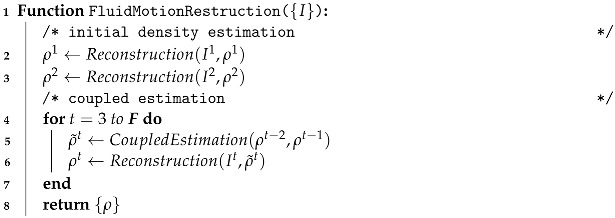


**Figure 2:**
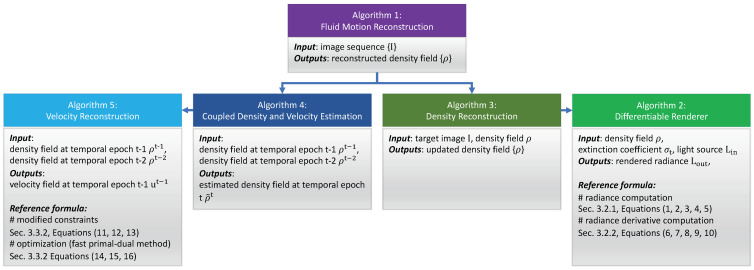
This figure provides a schematic diagram overviewing the relations between our proposed algorithms and the formulas presented in each subsection (Algorithm 2: Differentiable Renderer. Algorithm 3: Density Reconstruction. Algorithm 4: Coupled Density and Velocity Estimation. Algorithm 5: Velocity Reconstruction).

   To provide an overview of our algorithms and their relations to the formulas presented in each subsection, we include a schematic diagram in Figure 2. As depicted, our proposed method comprises five key algorithms. The core algorithm is Algorithm 1 (fluid motion reconstruction), which invokes the *Reconstruction* and *CoupleEstimation* algorithms. The *Reconstruction* algorithm reconstructs density fields from input images by leveraging our proposed differentiable renderer, to render images and compute derivatives. Equations (1)–(5), introduced in Section 3.2.1, govern the radiance calculation. Equations (6)–(10), presented in Section 3.2.2, enable radiance derivative computations.

**Algorithm 2:** Differentiable renderer

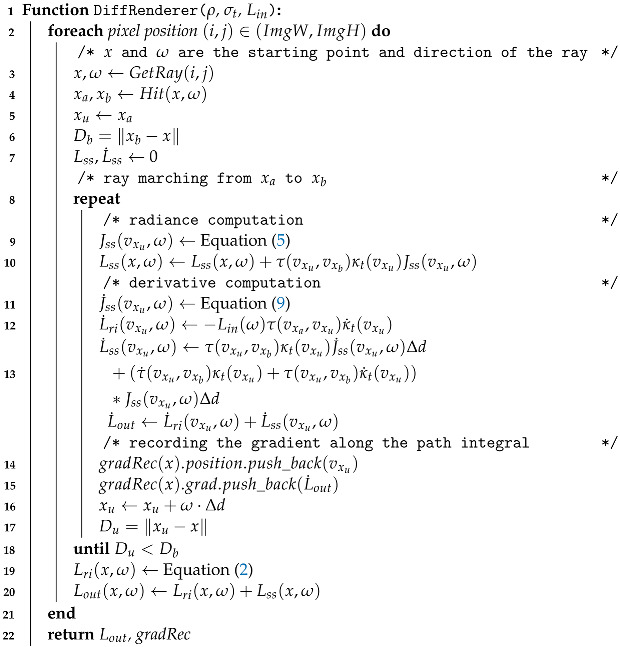



**Algorithm 3:** Density reconstruction

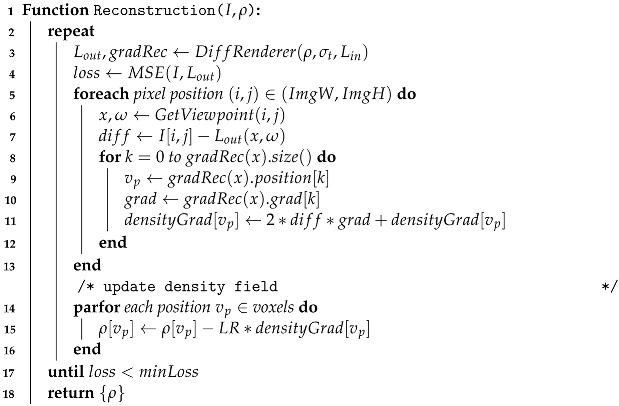



**Algorithm 4:** Coupled density and velocity estimation

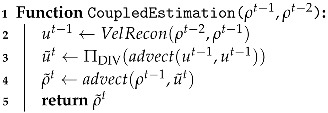



**Algorithm 5:** Velocity reconstruction

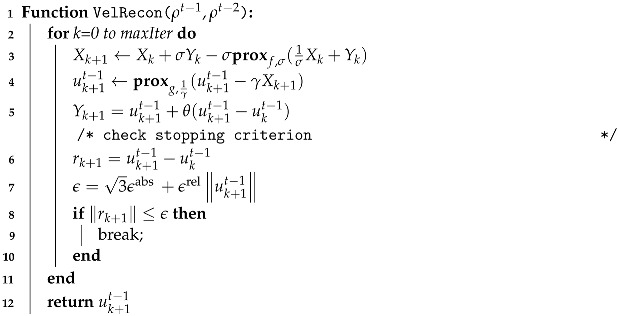



Algorithm 4 (coupled density and velocity estimation), introduced in Section 3.3, advects the reconstructed density fields from time step *t* − 1 and *t* − 2 to *t* while enforcing key physical constraints. Since it takes density fields as input, Algorithm 4 calls Algorithm 5 (velocity reconstruction) to estimate velocity fields from the previous two time steps’ density fields to perform advection. Velocity reconstruction is formulated as an optimization problem with Equations (11)–(13) (Section 3.3.2) as constraints. Algorithm 5 efficiently solves this using a fast primal-dual approach governed by Equations (14)–(16). Finally, the reconstructed velocity fields are advected to density fields from time step *t* − 1 to *t*. This procedure couples the reconstruction of densities and velocities in a physics-constrained manner for robust fluid motion estimation.

Further technical details concerning our proposed differentiable rendering framework and the coupled density and velocity estimation will be discussed below.

### 3.2. Differentiable Rendering

A fluid is a participating medium that can scatter or absorb light, altering its direction and intensity as it passes through. Modeling the behavior of light in a fluid can be difficult and computationally expensive, especially when multiple scattering events are involved. Many studies have addressed these challenges by assuming simplified lighting conditions or uncluttered backgrounds in the fluid reconstruction process. This approach helps minimize interference with accuracy. Our work follows this trend and posits the following hypotheses as a result:

- We assume that the single-scattering model is sufficient for rendering the rough shape of the fluid volume. Therefore, we ignore the radiance contribution of multiple-scattering radiance and emitted radiance.
- Our method is limited to pure volume scenes, which reduces the complexity of derivative computation caused by visibility.

We now depict the technical details of our differentiable rendering framework. First, we introduce the participating media rendering based on single scattering (Section 3.2.1). We then derive the differentiation form of the RTE (Section 3.2.2), which can be directly used to compute the radiance derivative with respect to density. We finally present the algorithm of fluid density reconstruction via our proposed differentiable renderer (Section 3.2.3 and Section 3.2.4).

#### 3.2.1. Participating Media Rendering

In order to lay the foundation for differentiating the radiative transfer equation (RTE), we will briefly review light transport in participating media and introduce the RTE based on the single-scattering model. When light passes through a participating media, it is attenuated along the direction ω and ultimately reaches the viewpoint *x* with the total radiance $L_{out}\left(x,\omega \right)$. As noted earlier, we assume that the light transport within the volume is governed solely by single scattering. This process can be mathematically modeled by the RTE, whose integral form is expressed as follows:

(1) $$\begin{array}{c} L_{out}\left(x,\omega \right)=L_{ri}\left(x,\omega \right)+L_{ss}\left(x,\omega \right). \end{array}$$

where $L_{out}$ comprises the reduced incident radiance $L_{ri}$ and single-scattering radiance $L_{ss}$. The reduced incident radiance $L_{ri}\left(x,\omega \right)$ describes the ambient light $L_{in}\left(\omega \right)$ that arrives directly at viewpoint *x* along direction ω with attenuation by the participating medium. The single-scattering radiance $L_{ss}\left(x,\omega \right)$ represents that the radiance of light has scattered only once before arriving at viewpoint *x* along the direction ω, which is computed by integrating radiance contributions along the corresponding view ray. Before detailing the expressions of the radiance, the following notations need to be predefined.

Let us denote the volume density of the participating medium at position *x* as ρ(x), and let the optical coefficient be defined as $\kappa_{i}\left(x\right)$, where $\kappa_{i}\in \left\{\kappa_{a},\kappa_{s},\kappa_{t}\right\}$ indicate the absorption, scattering, and extinction coefficient, respectively. The extinction coefficient is $\kappa_{t}\left(x\right)=\kappa_{a}\left(x\right)+\kappa_{s}\left(x\right)$, and the scattering albedo is $\Omega =\kappa_{s}\left(x\right)/\kappa_{t}\left(x\right)$. The expressions of $L_{ri}$ and $L_{ss}$ are as follows:

(2) $$\begin{array}{c} \begin{array}{cc} L_{ri}\left(x,\omega \right) & =L_{in}\left(\omega \right)\tau \left(x_{a},x\right), \end{array} \end{array}$$

(3) $$\begin{array}{c} \begin{array}{cc} L_{ss}\left(x,\omega \right) & =\int_{D_{b}}^{D_{a}}\tau \left(x_{u},x_{b}\right)\kappa_{t}\left(x_{u}\right)J_{ss}\left(x_{u},\omega \right)du, \end{array} \end{array}$$

where $D_{a}$ and $D_{b}$ are the distances from the viewpoint *x* to the volume boundary points $x_{a}$ and $x_{b}$, respectively. $x_{u}$ is an arbitrary position inside the participating medium volume. $\tau (x_{0},x_{1})$ is the transmittance from $x_{0}$ to $x_{1}$, which is computed as

(4) $$\begin{array}{c} \tau \left(x_{0},x_{1}\right)=e^{-\int_{D_{1}}^{D_{0}}\kappa_{t}\left(x_{u}\right)du}, \end{array}$$

where $x_{0}$ and $x_{1}$ are arbitrary positions inside the participating medium volume, and $D_{0}$ and $D_{1}$ are the distances from viewpoint *x* to $x_{0}$ and $x_{1}$, respectively. As both τ and $L_{ss}$ are expressed in continuous forms, they cannot be directly calculated in computers. We derive their discrete forms to facilitate computer program implementation in Section 3.2.2.

To compute $L_{ss}$, $J_{ss}$ is required. The single-scattering term $J_{ss}$ represents the reduced incident radiance, with its first scattering interaction occurring at the position $v_{p}$ of the voxel grid:

(5) $$J_{ss}\left(v_{p},\omega \right)=\frac{\Omega }{4\pi }\int_{S^{2}}L_{ri}\left(v_{p},\omega^{\prime }\right)p\left(\omega ,\omega^{\prime }\right)d\omega^{\prime },$$

where $S^{2}$ indicates a solid angle over the unit sphere. Due to Equation (5), we need to calculate an integration over the unit sphere $S^{2}$, while Monte Carlo (MC) integration provides a means for estimating $J_{ss}$. However, for more efficiency, we use distinct tricks for different illuminations. For environment map lighting, we utilize importance sampling to estimate $J_{ss}$ efficiently. For simple point or parallel lights, $J_{ss}$ can be directly calculated without MC as the lighting directions and radiance are known. Rather than relying on MC estimation for the complete differentiable rendering, we use it only to efficiently estimate $J_{ss}$ where needed. The phase function $p(\omega ,\omega^{\prime })$ describes the directional scattering distribution of the participating media.

The necessary formulas for forward rendering have been introduced. In the following section, we will derive the differential form of the RTE. This form can be integrated into the differentiable rendering process to calculate the derivative of the RTE directly.

#### 3.2.2. Differential Form of RTE

Here, we introduce the differentiation of the RTE based on the single-scattering model for computing the density derivative. The differentiation of Equation (1) can be expanded into two terms, namely the differentiation of the reduced incident radiance ${\dot{L}}_{ri}$ and the differentiation of the single-scattering radiance ${\dot{L}}_{ss}$. The differential form of the RTE is therefore

(6) $${\dot{L}}_{out}\left(x,\omega \right)={\dot{L}}_{ri}\left(x,\omega \right)+{\dot{L}}_{ss}\left(x,\omega \right).$$

Since our work only focuses on the fluid reconstruction task, the derivative ${\dot{L}}_{out}$ can be confined as ${\dot{L}}_{out}=\frac{dL_{out}}{d\rho }$ (w.r.t. density ρ only).

**Derivation of ${\dot{L}}_{ri}$**. As shown in Equation (6), the first component of the differential form of the RTE is ${\dot{L}}_{ri}$, which can be derived by differentiating $L_{ri}$ of Equation (2):

(7) $$\begin{array}{c} {\dot{L}}_{ri}\left(x,\omega \right)=L_{in}\left(\omega \right)\dot{\tau }\left(x_{a},x\right). \end{array}$$

In order to compute ${\dot{L}}_{ri}$, $\dot{\tau }$ is required. Differentiating the transmittance τ of Equation (4) yields

(8) $$\begin{array}{cc} \dot{\tau }\left(v_{x_{0}},v_{x_{1}}\right)= & -\tau \left(v_{x_{0}},v_{x_{1}}\right)\sum_{p=x_{0}}^{x_{1}}{\dot{\kappa }}_{t}\left(v_{p}\right)\Delta d, \end{array}$$

where ${\dot{\kappa }}_{t}=\sigma_{t}$, $\sigma_{t}$ is the extinction cross-section, $v_{p}$ is a voxel position of a uniform 3D voxel grid, and Δd is the length of the sampling step.

**Derivation of ${\dot{L}}_{ss}$**. The second component of the differential form of the RTE is ${\dot{L}}_{ss}$. Given the single-scattering radiance assumption, where light scattering occurs only once before arriving at viewpoint *x*, differentiating $L_{ss}$ of Equation (3) yields

(9) $$\begin{array}{cc} {\dot{L}}_{ss}\left(x,\omega \right)= & \sum_{p=x_{a}}^{x_{b}}\left(\dot{\tau }\left(v_{p},v_{x_{b}}\right)\kappa_{t}\left(v_{p}\right)+\tau \left(v_{p},v_{x_{b}}\right){\dot{\kappa }}_{t}\left(v_{p}\right)\right)J_{ss}\left(v_{p},\omega \right)\Delta d\\ \; & +\sum_{p=x_{a}}^{x_{b}}\tau \left(v_{p},v_{x_{b}}\right)\kappa_{t}\left(v_{p}\right){\dot{J}}_{ss}\left(v_{p},\omega \right)\Delta d. \end{array}$$

To compute ${\dot{L}}_{ss}$, ${\dot{J}}_{ss}$ is required. Differentiating $J_{ss}$ of Equation (5) yields

(10) $$\begin{array}{c} {\dot{J}}_{ss}\left(v_{p},\omega \right)=\frac{\Omega }{4\pi }\int_{S^{2}}{\dot{L}}_{ri}\left(v_{p},\omega^{\prime }\right)p\left(\omega ,\omega^{\prime }\right)d\omega^{\prime }. \end{array}$$

The derivation of Equation (10) requires taking the derivative of an integral. To accomplish this, we can utilize the Reynolds transport theorem [32,33], which is a widely used method for computing derivatives of hydrodynamic integral equations. However, since our work is concerned with purely participating media volumes, we can assume that $L_{ri}$ exhibits continuity in $S^{2}$, and thus the derivative of the interface can be disregarded.

#### 3.2.3. Differentiable Renderer

The cornerstone of our differentiable renderer lies in the radiance transport equation and its differential form. Unlike previous works, we derive an analytical differential expression for the radiance transport equation, thereby enabling the direct computation of density derivatives without the exclusive reliance on automatic differentiation. Our algorithmic approach is delineated in Algorithm 2, where we employ ray marching to both render images and compute the associated derivatives. Specifically, the algorithm calculates the final radiance $L_{\mathrm{out}}$ while simultaneously storing the requisite gradients for subsequent density field updates. Lines 9–13 of the algorithm illustrate that we execute both forward and backward passes concurrently. This dual-pass approach facilitates the reuse of $J_{ss}$ during the backpropagation phase.

It is worth noting that Equations (5) and (9), appearing in lines 9 and 11 of Algorithm 2, are essential for performing integration over the unit sphere. While a straightforward Monte Carlo method could be employed for this estimation, it would entail computationally expensive calculations. To mitigate this, we adopt specialized techniques tailored to different types of illuminations. As elaborated in Section 3.2.1, we utilize importance sampling for environment map illuminations and direct computation for point/parallel light illuminations when calculating $J_{ss}$.

Furthermore, as depicted in lines 14–15, gradRec is used for recording the gradients associated with the sampling positions. Rather than saving gradients for all voxels, we implement a sparse structure gradRec that only stores gradients along sampled ray paths. When marching rays through the volume, we push gradients onto the stack as intersections occur along the ray (Algorithm 2 lines 14–15). When a density field update finishes (Algorithm 3), the stack is popped, releasing memory. Our optimized approach reduces memory usage from $O(N^{3})$ to O(RN) for *R* rays of length *N*. We only maintain gradients along active ray paths rather than the entire volume grid. These computational strategies, in conjunction with our analytical radiance derivatives, culminate in a highly efficient differentiable renderer, thereby facilitating fluid density optimization with enhanced computational efficacy.

#### 3.2.4. Density Reconstruction

Building upon our proposed differentiable rendering framework, we present the reconstruction of the density field using our framework and provide details in Algorithm 3. We begin by computing the loss function of the target and primal images, followed by reading the density gradient from the recorded sampling positions of the generated ray. The gradients of density with respect to the loss value are computed and saved in the volume data densityGrad. Finally, we iterate through and update each voxel value of the density field, and the memory of the structure gradRec and densityGrad are released.

### 3.3. Coupled Density and Velocity Estimation

Most previous works for fluid reconstruction directly updated the fluid density in each time step using tomographic methods from input video sequences. While this works well for systems with a large number of cameras, the reconstruction quality drops significantly when the number of cameras (i.e., projections) reduces, making the tomography problem formulation ill-posed. To solve this ill-posed problem and improve the reconstruction quality, we apply a coupled estimated strategy, which makes the efficient reconstruction of realistic and smooth fluid flow possible. A possible solution is coupled density and velocity updating, which was developed by Eckert et al. [4], yet their method was extremely time-consuming and impractical, with an average reconstruction time of up to 50–60 min per frame. We therefore propose a simplified strategy for coupled density and velocity estimation that is highly efficient, making interactive fluid motion reconstruction possible.

#### 3.3.1. Coupled Estimation

Our approach is to predict the density field ${\tilde{\rho }}^{t}$ for the current time step *t* based on the given states of time *t* − 1 and time *t* − 2. The details of our coupled density and velocity estimation are depicted in Algorithm 4. To proceed, we first estimate velocity $u^{t-1}$ based on the densities of time *t* − 1 and time *t* − 2 according to Algorithm 5. We then predict the velocity and density for the current time step *t* based on physical priors, i.e., the density and velocity transport over the time steps and the incompressibility of the velocity. Note that incompressibility is ensured by projecting the velocities onto the space of divergence-free velocities, called $\Pi_{\mathrm{DIV}}$. We advect velocity $u^{t-1}$ with itself and ensure its incompressibility to create a velocity guess ${\tilde{u}}^{t}$. The density guess ${\tilde{\rho }}^{t}$ is created by advecting the density $\rho^{t-1}$ forward with the velocity guess ${\tilde{u}}^{t-1}$ (lines 3–4 of Algorithm 4).

#### 3.3.2. Velocity Reconstruction

While the last two steps shown in lines 3–4 of Algorithm 4 are similar to those in [4], our velocity reconstruction process is different. Notably, we do not apply *depth regularization* to gain control over the motion in depth. Because this regularized term breaks the sparse property of the matrix, the optimization problem cannot be solved efficiently.

To improve the efficiency of velocity reconstruction, we propose a modified constraint as follows:

(11) $$\begin{array}{c} \begin{array}{cc} \min_{u_{t-1}}f\left(u^{t-1}\right)= & {∥\frac{\rho^{t-1}-\rho^{t-2}}{\Delta t}+u^{t-1}\cdot \nabla \rho^{t-1}∥}^{2}\\ \; & +E_{smooth}\left(u^{t-1}\right)+E_{kinetic}\left(u^{t-1}\right) \end{array} \end{array}$$

(12) $$\begin{array}{c} \begin{array}{cc} \;\mathrm{subject}\;\mathrm{to}\;\;\nabla \cdot u^{t-1}= & 0. \end{array} \end{array}$$

In Equation (11), the first component is the constraint from an optical flow problem $\frac{\partial \rho }{\partial t}+\nabla \cdot \left(\rho u\right)=0$. In order to further confine our solution, we add both smoothness and kinetic energy (Tikhonov) regularized terms to the velocity [34]. These regularized terms are expressed as

(13) $$\begin{array}{cc} E_{smooth}\left(u\right)= & \alpha {∥\nabla u∥}^{2},\\ E_{kinetic}\left(u\right)= & \beta {∥u_{t-1}∥}^{2}, \end{array}$$

where α,β are the weights of these regularized terms, respectively. $E_{smooth}\left(u\right)$ is a smoothness regularizer controlled by weight α. It penalizes large changes/gradients in the velocity field by minimizing the L2 norm of ∇u. This smoothness constraint helps reduce noise and discontinuities in the estimated velocities. $E_{kinetic}\left(u\right)$ is a kinetic energy or Tikhonov regularizer weighted by β. It minimizes the L2 norm of the velocity values themselves. This constraint discourages large velocity magnitudes and excessive motion. Together, these regularization terms help confine the velocity solution space to plausible flows that are smooth and controlled in magnitude. The relative weights α and β allow the balancing of the smoothing and kinetic energy constraints as needed for the data.

In order to compute the velocity, as described in Equations (11) and (12), we make use of the fast primal-dual method (PD) for convex optimization as introduced by Chambolle and Pock [35]. Instead of solving for the whole complex optimization problem at once, we split the complex optimization problem into two manageable components, where proximal operators act as efficient solvers for each subproblem. Iterative variable updates ensure that the solution converges to the optimal value of the problem in Equations (11) and (12). The simplified PD updates are given by

(14) $$\begin{array}{cc} X_{k+1}= & X_{k}+\sigma Y_{k}-\sigma {\mathrm{prox}}_{f,\sigma }\left(\frac{1}{\sigma }X_{k}+Y_{k}\right),\\ u_{k+1}^{t-1}= & {\mathrm{prox}}_{g,\frac{1}{\gamma }}\left(u_{k+1}^{t-1}-\gamma X_{k+1}\right),\\ Y_{k+1}= & u_{k+1}^{t-1}+\theta \left(u_{k+1}^{t-1}-u_{k}^{t-1}\right), \end{array}$$

where {σ,γ,θ} are parameters that affect convergence; *k* is the iteration number; X,Y are helper variables; and prox is the proximal operator for each subproblem. *f* of ${\mathrm{prox}}_{f,\sigma }$ targets Equation (11), where velocity $u^{t-1}$ is unknown, and the object function *f* (Equation (11)) is solved by least squares. The optimization equation of ${\mathrm{prox}}_{f,\sigma }$ is given by

(15) $${\mathrm{prox}}_{f,\sigma }\left(\xi \right)=argmin_{u^{t-1}}f\left(u^{t-1}\right)+\frac{\sigma }{2}∥u^{t-1}-\xi ∥,$$

where ξ is a generic variable. The proximal operator ${\mathrm{prox}}_{g,\frac{1}{\gamma }}$ targets Equation (12), and this constraint assumes that the velocity field is incompressible:

(16) $${\mathrm{prox}}_{g,\frac{1}{\gamma }}\left(\xi \right)=\Pi_{\mathrm{DIV}}\left(\xi \right).$$

The process of velocity reconstruction is shown in Algorithm 5. Lines 3–5 are the PD updates, and lines 6–10 check the convergence of the iterative computation.

### 3.4. Implementation Details

We implemented our proposed approach using a combination of Python and C++. The differentiable renderer was developed in Python, leveraging the Taichi programming language [36,37] for parallelization and high-performance computing on GPUs. Taichi was chosen due to its excellent compatibility with existing deep learning frameworks, allowing integration with neural networks or other models. Additionally, Taichi provided high-efficiency parallel primitives and differentiable programming constructs that significantly accelerated the core computations in our renderer compared to standard Python.

The coupled density and velocity estimation component was implemented in C++ for computational efficiency and numeric precision. We interfaced with this C++ module from the Python renderer using the pybind11 library. Pybind11 enabled us to seamlessly pass data between the Python and C++ code.

We evaluated our method on a workstation with an Intel Xeon E5-2620 v4 CPU and an Nvidia GeForce RTX 2080 Ti GPU. All results and timings provided were obtained by executing the code on the GPU hardware, leveraging parallelized implementations for performance.

## 4. Result

### 4.1. Evaluation

We conducted a rigorous validation of our method through both qualitative visualizations and quantitative analyses. Our validations focused primarily on the claimed contributions of the proposed differentiable renderer and reconstruction framework. As the accuracy of the estimated gradients and computational performance are critical metrics for evaluating a differentiable renderer, we first validated these two aspects. Subsequently, we conducted ablation studies to assess the reconstruction capabilities of our differentiable renderer and the efficacy of the transport constraints. Furthermore, given the significant impact of the estimated velocity on reconstructed fluid motions, we analyzed comparisons between our velocity estimates and the ground-truth data. Finally, we compared our approach against previous methods through both qualitative visualizations and quantitative efficiency metrics.

**Figure 3:**
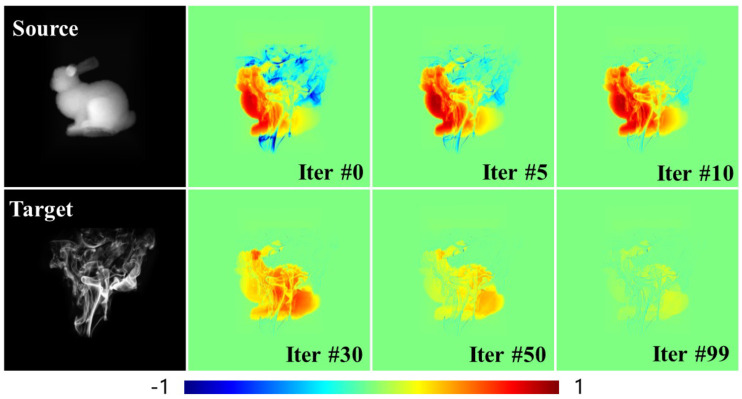
Derivative analysis of density optimization. The initial volume was configured in the shape of a rabbit, with the density field optimized to match a target smoke image. The derivative images show an increasing density in blue and decreasing density in red. This color mapping accurately captures the evolution of the derivatives throughout the entire density optimization process.

**Validation of gradient computation.** To validate the effectiveness of our proposed differentiable renderer, we optimized a density field and analyzed the evolution of the calculated derivatives. The initial density configuration was in the shape of a rabbit, which was optimized by updating the density field based on a target smoke image. The image sequence in Figure 3 shows the images derived throughout the optimization iterations.

Initially, the density inside the rabbit shape needed to decrease, while the density in the smoke region had to increase to match the target. Since the smoke density was thinner than the rabbit density, the derivatives for the smoke density diminished faster, disappearing after around 30 iterations. In contrast, significant derivatives for the rabbit density persisted from iterations 0 to 50. By iteration 99, the optimization had converged to the target image. Overall, Figure 3 demonstrates that the density field derivatives followed the expected variations throughout the optimization process.

**Performance validation of our differentiable renderer.** Since our differentiable renderer significantly differed from previous methods based on differential volumetric path tracing in implementation, different factors impacted its performance. Previous physics-based differentiable renderers heavily relied on the Monte Carlo method to estimate the radiance and its derivatives. Hence, these methods’ performance was affected by the number of samplings. Unlike these methods, the volumetric data size significantly impacted our renderer’s performance. To characterize this relationship, validations were performed on the rabbit scene in Figure 3 under four resolutions: $32^{3}$, $64^{3}$, $128^{3}$, and $256^{3}$ voxels. Table 1 presents the average runtime and GPU memory usage across 100 iterative optimizations at different volumetric data resolutions. As anticipated, the runtime of our renderer demonstrated an increasing trend with higher-resolution volumetric data. The recorded runtimes for the resolutions of $32^{3}$, $64^{3}$, $128^{3}$, and $256^{3}$ voxels were 9.4 ms, 17.6 ms, 82.4 ms, and 545.1 ms, respectively. Despite the increase in runtime, our method maintained real-time performance even at higher resolutions, which could be crucial for interactive applications and dynamic scenes.

The memory usage of our differentiable renderer was another essential aspect of its performance. Similar to the runtime analysis, we observed a direct correlation between the resolution of the volumetric data and the GPU memory usage. As the resolution increased, so did the memory requirement. The memory usages for the resolutions $32^{3}$, $64^{3}$, $128^{3}$, and $256^{3}$ voxels were 612 MB, 648 MB, 851 MB, and 2813 MB, respectively. This increase was consistent with the greater amount of data that needs to be processed and stored in higher-resolution volumetric data. Despite this growth, our method remained memory-efficient even at the highest resolution, which was a testament to its practical viability for a wide range of applications.

**Figure 4:**
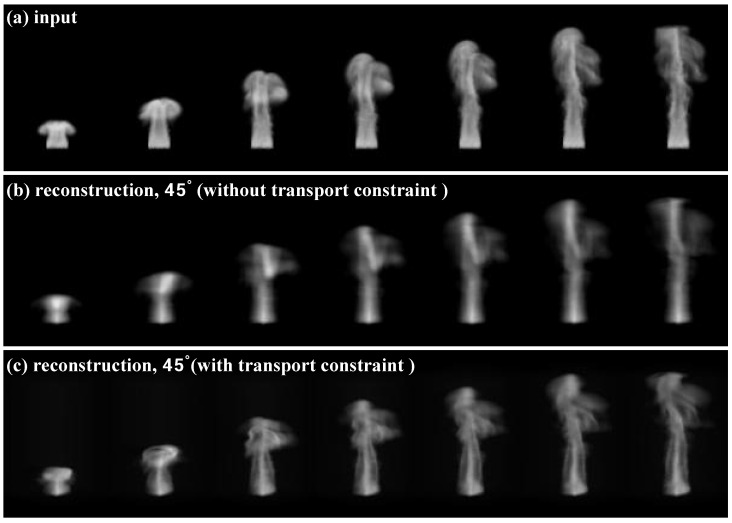
Validation of the physical constraint. (**a**) The input image sequence used to constrain the shape from orthogonal views. (**b**) The reconstructed results without the transport constraint. (**c**) The proposed constraint. We observed that adding the transport constraint significantly improved the reconstruction quality and temporal consistency.

**Evaluation of density reconstruction.** To evaluate the reconstruction accuracy of our proposed method, we conducted an experiment using synthetic fluid motion data. The ground-truth fluid flows were generated using the *Mantaflow* simulator [38]. The density fields were then rendered into a monocular video sequence using *Mitsuba 3* [39]. This synthetic video served as the target for guiding the reconstruction using our approach. To enhance the shape constraints, we re-used the monocular video to constrain the reconstruction from orthogonal angles (e.g., angle $0^{\circ }$ and $90^{\circ }$). As shown in Figure 4, we conducted an ablation experiment to study the effectiveness of the proposed *coupled density and velocity estimation component*. Qualitatively, the physically constrained result in Figure 4c more faithfully captured the true fluid evolution compared to the unconstrained reconstruction in Figure 4b. The results demonstrated that our proposed framework could accurately reconstruct fluid motions from monocular video while maintaining physically consistent temporal evolutions.

**Figure 5:**
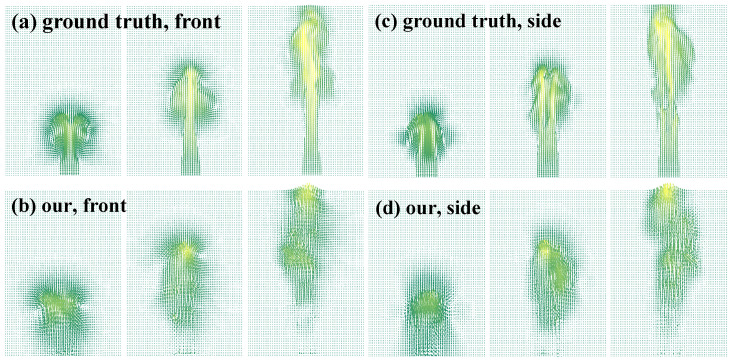
Validating the velocity estimation. We compared center-slice velocities from our estimation to the ground truth at frames t=70,110, and 147 along the front and side views. The results exhibited some bias near the base, as no specialized inflow treatment was implemented. However, overall, the estimated velocities reasonably matched the ground truth, suggesting that our method could effectively and reliably characterize the fluid flow dynamics.

**Evaluation of the velocity estimation.** The evaluation of the velocity estimation is of utmost importance in reconstructing fluid motions, as it significantly impacts the reconstruction quality and temporal consistency of fluid motions. We compared estimated velocity fields obtained from reconstructing Figure 4 with the ground truth. As Figure 5 shows, the estimated velocity was somewhat minimal at the base because no specialized processing was implemented for the inflow zones. Despite the inherent challenges in precisely estimating inflow velocity from only a monocular video and transport constraints, the overall velocity field could still be coarsely estimated to a reasonable degree. These findings validated the feasibility of estimating fluid velocities from limited visual information while preserving efficiency. In applications where computational efficiency is not the primary concern, one potential area of investigation could be devising techniques to better model the inflow velocity profile, which would likely improve estimated accuracy.

**Figure 6:**
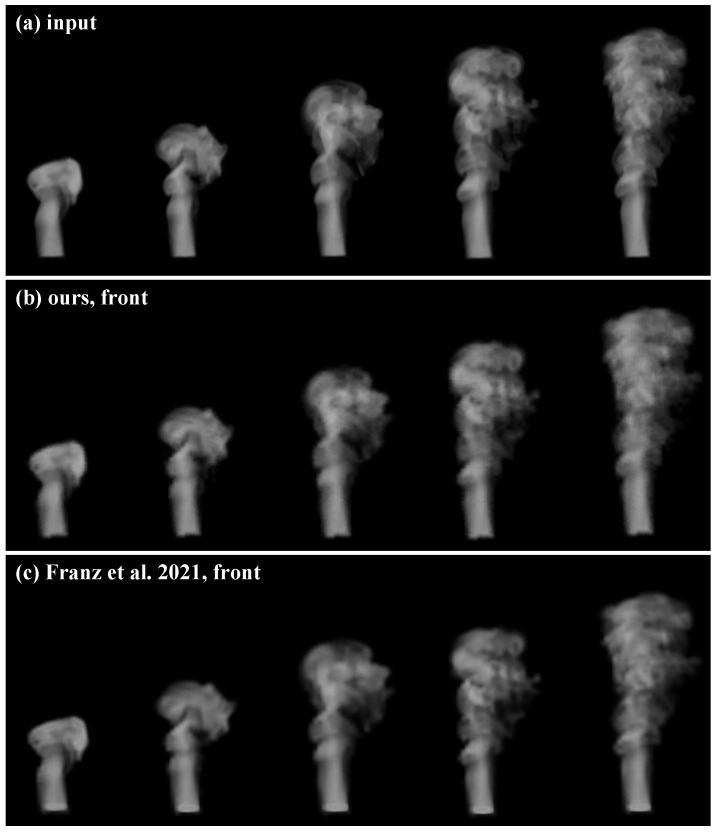
Orthogonal view (angle 0${}^{\circ }$ and angle 90${}^{\circ }$) reconstruction. The results demonstrate that our approach could produce highly realistic fluid motion that matched the ground truth closely. Our comparison with Franz et al. [5]’s method revealed that our method’s reconstructed ability was comparable to theirs.

**Comparison to previous work.** While we demonstrated our method’s fluid motion reconstruction capabilities above, further comparative evaluations were essential to fully validate its abilities against previous approaches. To this end, we conducted fluid motion reconstruction from the same initial configuration using both our method and Franz et al.’s approach [5]. Our method followed similar overall workflows to Franz et al.’s approach but had key differences in the following critical aspects: First, we proposed an efficient differentiable renderer for participating media, which was successfully integrated into our framework. Second, our method utilized an improved joint density and velocity estimation strategy. These improvements aimed to enable efficient and temporally consistent fluid motion reconstruction. As depicted in Figure 6, both our method and Franz et al.’s method achieved a high similarity between visualizations and the ground truth. This demonstrated that our framework had a comparable fluid motion reconstruction capability to Franz et al.’s method.

Furthermore, run-time efficiency is an important indicator to evaluate a reconstruction framework. To validate the efficiency of our proposed framework, we compared the average runtime of our method against Franz et al.’s method for reconstructing fluid densities from video inputs. As shown in Table 2, we evaluated three volumetric resolutions of 64 × 96 × 64, 128 × 192 × 128, and 256 × 288 × 256 voxels.

For the 64 × 96 × 64 resolution, our complete reconstruction time per frame was 6.13 s, over 59× faster than Franz et al.’s 363 s. More significantly, our differentiable rendering time was just 0.08 s, demonstrating a speedup of over 870× compared to their 69.6 s for this key computational stage.

For the higher resolutions of 128 × 192 × 128 and 256 × 288 × 256, our method outperformed Franz et al.’s by factors of approximately 172 and 173, respectively, in terms of average reconstruction time. Furthermore, our differentiable rendering stage exhibited speedup factors of around 2655 and 1676 compared to Franz et al.’s methodology.

### 4.2. Reconstruction from Real-World Data

**Figure 7:**
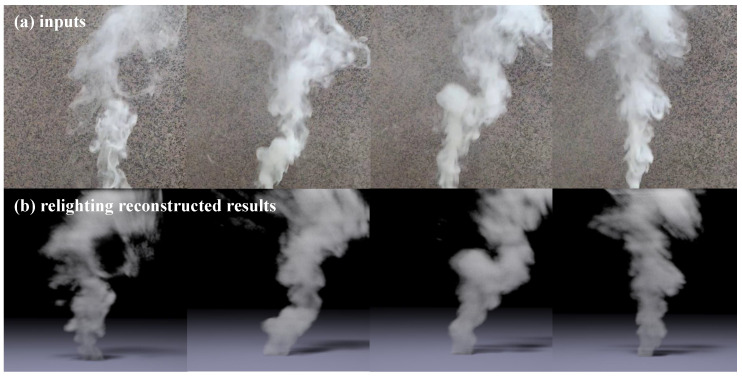
Reconstruction from real-world data. We utilized a monocular video captured from real-world fluid flows as input into our method, reconstructing at a volumetric resolution of 64 × 96 × 64. As illustrated in (**b**), the reconstructed flows rendered via *Mitsuba 3* demonstrated that our proposed approach could reconstruct physically plausible fluid motions from real-world video.

Reconstructing fluid flows from real-world video captures presents significant challenges due to ambient interference and computationally intensive processing requirements. Previous approaches have required several minutes per frame for reconstruction, severely limiting applicability in interactive contexts. Our proposed method aimed to address this limitation by enabling the efficient modeling of fluid motions from monocular videos of real fluids. To validate the practical applicability of our method, we reconstructed fluid motions from a monocular video capturing real fluid flows from a fixed viewpoint. As depicted in Figure 7, the reconstructed results were rendered by *Mitsuba 3* [39] from a perspective offset by 20 degrees away from the frontal view. Our results demonstrated that our method could reconstruct fluid motions realistically with high shape fidelity compared to the input video, even for complex backgrounds. This showcased the potential of our method as a useful and efficient tool for artistic creation and content generation.

## 5. Conclusions

We introduced a novel method to efficiently reconstruct fluid density fields from monocular video data. The key contribution was our newly proposed efficient differentiable rendering framework for participating media and the improved transport constraints. Our approach outperformed previous methods by considering both computational efficiency and visual quality. Looking ahead, worthwhile future work includes reducing current limitations, such as incorporating the differentiable rendering of surface models and developing advanced sampling strategies to further improve efficiency. Overall, this work represents an important advance in performing monocular video-based fluid reconstructions, providing a valuable new tool for digital content creation.

## Figures and Tables

**Table 1:** Performance validation at different volumetric data resolutions. The average runtime and GPU memory utilization across 100 optimization iterations are presented for varying volumetric data resolutions. We observed increasing runtimes and memory usage with higher resolutions. However, our differentiable renderer maintained real-time performance and memory efficiency even at elevated resolutions.

Volumetric Resolution	Average Runtime (ms)	Memory Usage (MB)
$32^{3}$	9.4	612
$64^{3}$	17.6	648
$128^{3}$	82.4	851
$256^{3}$	545.1	2813

**Table 2:** A comparison of efficiency with Franz et al.’s works. “Time” indicates the average reconstruction time per frame.

Method	Resolution	DR Time	Total Time
Franz et al. [5]	64 × 96 × 64	1.16 m	6.06 m
	128 × 192 × 128	5.31 m	25.2 m
	256 × 288 × 256	21.8 m	61.6 m
Our method	64 × 96 × 64	0.08 s	6.13 s
	128 × 192 × 128	0.12 s	8.75 s
	256 × 288 × 256	0.78 s	21.31 s

## Data Availability

Not applicable.
